# How do children with Tourette’s syndrome and their caregivers live with the disorder? A systematic review of qualitative evidence

**DOI:** 10.3389/fpsyt.2022.992905

**Published:** 2022-09-29

**Authors:** Hyo-Weon Suh, Seok-In Yoon, Sunggyu Hong, Hyun Woo Lee, Misun Lee, Jong Woo Kim, Sun-Yong Chung

**Affiliations:** ^1^Department of Neuropsychiatry, College of Korean Medicine, Kyung Hee University, Seoul, South Korea; ^2^Department of Neuropsychiatry, Kyung Hee University Korean Medicine Hospital at Gangdong, Seoul, South Korea; ^3^Industry-Academic Cooperation Foundation, Kyung Hee University, Seoul, South Korea

**Keywords:** tic disorders, Tourette syndrome, systematic review, qualitative research, meta-aggregation

## Abstract

**Background:**

Tourette’s syndrome (TS) is a childhood neurodevelopmental disorder characterized by sudden, repetitive, involuntary, and irregular muscle movement and vocalization. Recently, non-pharmaceutical methods, such as behavioral therapy, psychotherapy, and deep brain stimulation, have been introduced as alternatives to pharmacological treatment for TS. This study aimed to systematically review and synthesize qualitative evidence on the experiences of children with TS and their caregivers. A meta-synthesis of qualitative evidence could help provide a comprehensive understanding of the challenges experienced by children with TS and their caregivers with the aim of providing more effective treatment and services for them.

**Materials and methods:**

A systematic search was conducted using MEDLINE/PubMed, EMBASE, Cumulative Index to Nursing and Allied Health Literature, PsycARTICLES, and three Korean databases (Korean Medical Database, Research Information Sharing Service, and ScienceON) in July 2021. Studies were included if they collected and analyzed qualitative data from children with tic disorder or TS, or their caregivers. Qualitative research findings on the experiences and perspectives of children with TS and their caregivers were critically appraised and synthesized using the Joanna Briggs Institute methodology.

**Results:**

Eight eligible studies were included. The findings from these studies (i.e., themes or subthemes of qualitative research) were aggregated into categories (a group of similar findings) and synthesized findings (a group of categorized findings). Ultimately, the 60 findings were aggregated into 15 categories. Finally, four synthesized findings were derived from the 15 categories: (i) continuation of challenging daily life, (ii) denying that TS causes emotional distress, (iii) accepting and understanding TS as part of oneself, and (iv) looking to the future.

**Conclusion:**

Children with TS and their caregivers experience physical and psychological distress and social deprivation. Avoiding and suppressing TS causes secondary distress such as guilt. However, seeking social support and accepting the disorder reduce the distress caused by symptoms and lays the foundation for later growth. Even in the face of adversity, children with TS and their caregivers find personal value and acquire a more open and optimistic attitude toward life. This review shows that acceptance-based therapy and social support should be provided for the treatment and management of TS.

## Introduction

Tic disorder is a childhood neurodevelopmental disorder characterized by sudden, repetitive, involuntary, and irregular muscle movements and vocalizations (1). Motor and vocal tics generally disappear spontaneously within 1 year. However, if both types of tics persist for more than 1 year, the person is diagnosed with Tourette’s syndrome (TS) (1). This disorder was first reported by Gilles de la Tourette in France in 1885. Since then, studies have been conducted on the symptoms, causes, treatment, and prognosis of TS (2–4).

According to a meta-analysis (5), the prevalence of transient tic disorder in children was 2.99%. The prevalence of TS in children was 0.77% (1.06% in males and 0.25% in females). In addition, 88% of patients with TS have comorbidities (6). The most common disorders are attention deficit hyperactivity disorder, followed by obsessive-compulsive disorder (OCD) (7). Anger control and sleep problems are positively correlated with comorbidities in patients with TS (7).

According to a long-term follow-up study, patients with TS gradually worsen after the initial onset, reaching a peak at 10 years old. The symptoms of TS gradually decrease after adolescence (3). However, comorbidities, such as OCD become more severe at a later age and have been found to persist longer than TS (8).

Stigma is one of the main problems faced by children with TS that needs attention not only at individual level but also at social level (9). Children with TS experience social stigma, such as discrimination and bullying, but also self-stigma such as self-degrading (10). In addition, caregivers of children with TS experience courtesy stigma and feel guilty for their children and experience self-blame (10). As such, stigma negatively affects both the patient and the caregiver. Therefore, investigating the experiences of children and their caregivers with respect to stigma may help address the mental health problems associated with TS (11, 12).

Various methods, such as drugs, behavioral and psychotherapy, and deep brain stimulation, are used to treat TS. Drugs are the oldest treatment for TS (13). However, drugs for TS have limitations in that complex factors, including the side effects of drugs and patient characteristics such as age and comorbidities, must be considered (2). Due to these limitations, non-pharmacological treatments, such as behavioral and psychotherapy and deep brain stimulation, have recently become promising alternatives to treat TS. Evidence-based guidelines for non-pharmacologic therapies are being introduced in Europe and Canada (14, 15). Wile and Pringsheim (16) suggest that behavioral and psychotherapy, such as the Comprehensive Behavioral Intervention for Tics, can help to effectively improve the severity of tics.

Psychotherapy is required not only for children with TS but also for their caregivers. Parents of children with TS experience considerable stress. Parents of children with TS have higher parental stress than parents of children without TS (17). Parents of children with TS have a higher burden on caregivers and less psychological health than parents of children with asthma (18). In addition to parenting stress, parents of children with TS are concerned that their children will not achieve adequate achievement in the context of academic and social relationships (19, 20), and experience difficulties due to a lack of understanding from schools and society (21).

Recently, qualitative research on the experiences of children with TS and their caregivers has increased. Qualitative research can provide valuable insight into an individual’s lived experience and can help to explore complex phenomena that are difficult to understand only with quantitative research in the clinical field (22). Therefore, a comprehensive review of qualitative research on the experiences of children with TS and their caregivers can be a valid basis for comprehensively understanding the challenges experienced by patients and caregivers due to TS and providing effective treatment and services for them.

This study aimed to systematically review and synthesize the evidence of qualitative research on tic disorder or TS to explore the characteristics of TS and understand the experiences and perspectives of children with TS and their caregivers.

## Materials and methods

### Search strategy

A systematic search was conducted in MEDLINE/PubMed, EMBASE, Cumulative Index to Nursing and Allied Health Literature (CINAHL), PsycARTICLES, and three Korean databases [Korean Medical Database (KMbase), Research Information Sharing Service (RISS), and ScienceON] on July 2, 2021. The search terms used were “Tics,” “Tic Disorders,” “Tourette Syndrome,” “Qualitative Research,” “Qualitative,” and “Interview.” There were no limitations to the search in terms of language or publication date. Further details of the search protocol are presented in Supplementary Table 1.

### Study selection

The inclusion and exclusion criteria were developed and organized using the Sample, Phenomenon of Interest, Design, Evaluation, Research type (SPIDER) tool (23). The sample included children with tic disorder or TS, or their caregivers. The phenomena of interest were the experiences of tic disorder or TS and the perspectives of individuals challenged by the disorder. Studies were included if they were designed to collect data through surveys, interviews, or observations, and data were analyzed using qualitative research methodologies, such as ethnography, grounded theory, phenomenology, and consensual qualitative research. Both qualitative and mixed-methods studies were included. However, studies with unsupported results or no themes were excluded. Quantitative studies were also excluded.

Five researchers (H-WS, S-IY, SH, HL, and ML) independently screened and excluded studies that were not qualitative or were not related to TD or TS by evaluating their titles and abstracts. Then, the full text of potential articles was retrieved to consider eligibility. Disagreements were resolved by discussion.

### Quality assessment

Methodological quality was assessed using the Joanna Briggs Institute (JBI) critical appraisal checklist for qualitative research, which is a 10-item standardized critical appraisal instrument (24). Five items (numbers 2, 3, 4, 6, and 7) assessed the quality of the methodology and were used to determine the ConQual-dependability ranking (25). The other five items assess congruency from a philosophical perspective, as well as the methodology, interpretation of results, presentation of participants, ethics, and the conclusion.

The ConQual-credibility ranking represents the congruency between the authors’ interpretation and the study data. Each finding was assessed as “unequivocal (U),” “credible (C),” or “not supported (N)” based on the extent to which they were supported by illustrations from participants’ voices. Unequivocal findings are supported by evidence beyond reasonable doubt that is not open to challenge. Credible findings are interpretive and, therefore, open to challenges. Findings that are classified as “not supported” were not supported by the data. Therefore, they were not included in the meta-aggregation. Two researchers (H-WS and S-IY) independently assessed the studies and any discrepancies were discussed.

### Data extraction and data synthesis methods

We used the JBI method to extract and synthesize the review findings (24). JBI, a research institute established in 1996, has conceptualized the clinical value of qualitative systematic reviews and provided systematic tools and procedures to promote evidence-based medicine for qualitative research (24, 25). To determine the characteristics of the included studies, we extracted the year of publication, first author, country, study objectives, characteristics and number of participants, methodologies and methods. To synthesize qualitative evidence, we extracted themes, subthemes, and supportive quotations from the original studies. If a study qualitatively investigated the effects of an intervention, we only extracted the findings related to the phenomena of interest and excluded those related to the effects or responses to treatment.

The data were extracted by two authors (S-IY and ML) and verified by another researcher (H-WS). To clarify the meaning of the extracted findings, the authors (S-IY) thoroughly reviewed all findings and quotations. The findings were categorized according to similarity by one author (S-IY), and then a consensus was reached with another researcher (H-WS). Finally, the synthesized findings were drawn from the resulting meta-aggregation of categories.

### Confidence of the synthesized findings

The level of confidence in the synthesized findings was assessed and evaluated using the ConQual approach (25), which is based on dependability and credibility. Dependability refers to the consistency and stability of qualitative findings, which corresponds to reliability in quantitative studies. Credibility is the extent to which qualitative findings represent the truth, which corresponds to internal validity in quantitative studies. At the start of the ConQual assessment, qualitative research starts at “high,” and expert opinion starts at “low.” Downgrading of dependability occurred when the included studies did not meet at least four of the five criteria for dependability, and downgrading of credibility occurred when there was a mixture of unequivocal and equivocal findings. The confidence of estimates of the synthesized findings for both dependability and credibility leads to an overall ranking for each synthesized finding, which ranges from “high” to “very low” (“high,” “moderate,” “low,” and “very low”).

## Results

### Study and participant characteristics

**FIGURE 1:**
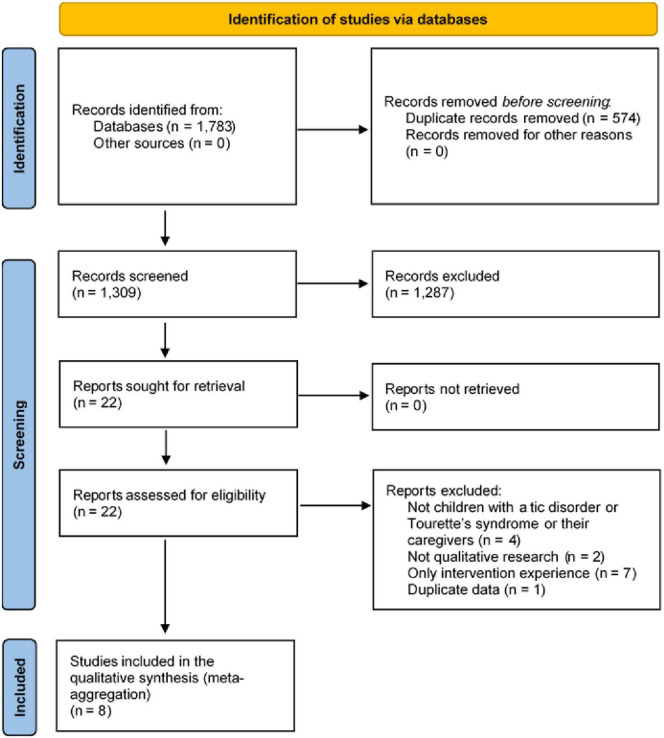
PRISMA flow chart.

The electronic search yielded 1,783 articles. A total of 1,309 potential articles remained after the duplicates were removed. After screening the titles and abstracts, 22 articles were selected for full-text analysis. Finally, eight studies (26–33) were included and synthesized in this review (Figure 1). The overall characteristics and extracted findings of the included studies are summarized in Tables 1, 2, respectively.

**TABLE 1:** Characteristics of the included studies.

No.	Study	Country	Objective	Participants (*n*)	Methodology	Methods
1	Kim and Tak (26)	South Korea	To develop a substantive theory on the psychological stress and coping strategies of parents of children older than 16 years old with Tourette’s syndrome	Parents of children with Tourette’s syndrome (*n* = 10)	Grounded theory	Unstructured interview
2	Travis and Juarez-Paz (27)	USA	To investigate supportive communication experienced by caregivers of children with Tourette’s syndrome	Caregivers of children with Tourette’s syndrome (*n* = 11)	Grounded theory	Semi-structured interview
3	Lee et al. (28)	Taiwan	To describe the essence of the self-experience of adolescents with Tourette’s syndrome in the context of peer interaction	Adolescents with Tourette’s syndrome (*n* = 12)	Phenomenology	Open-ended in-depth interview
4	Rindner (29)	USA	To describe how children and adolescents with Tourette’s syndrome experience embarrassment and determine which self-identified empowerment strategies children and adolescents with Tourette’s syndrome use to overcome the embarrassment	Participants with Tourette’s syndrome between nine and 17 years old (*n* = 18)	Phenomenology	Semi-structured interview
5	Ludlow et al. (30)	UK	To further explore the daily experiences of parents raising a child with Tourette’s syndrome	Parents or caregivers of a child with Tourette’s syndrome between seven and 17 years old (*n* = 15)	Thematic analysis	Semi-structured interview
6	Edwards et al. (31)	Canada	To explore how young people with Tourette’s syndrome understand their symptoms and are impacted by their condition and investigate what young people perceived as the most useful advice they have received from healthcare professionals regarding Tourette’s syndrome, and what advice they would share with newly diagnosed peers	Participants with Tourette’s syndrome between six and 17 years old (*n* = 13)	Thematic analysis	Semi-structured interview
7	Cutler et al. (32)	UK	To investigate the impact of Tourette’s syndrome on young people’s quality of life	Participants with Tourette’s syndrome between seven and 17 years old (*n* = 11)	Thematic analysis	Focus group interview
8	Lee et al. (33)	Taiwan	To explore and describe the experience of social adjustment of adolescents with Tourette’s syndrome in Taiwan	Adolescents with Tourette’s syndrome (*n* = 16)	Phenomenology	Semi-structured interview

**TABLE 2:** Findings of the included studies.

Study	Dependability	Numbered findings	Credibility
Kim and Tak (26)	5	1. Feeling like everything is falling apart	U
		2. Experiencing extreme stress	U
		3. Facing constant anxiety	U
		4. Can get worse again	U
		5. People’s prejudiced gaze	U
		6. Discovering improvement	U
		7. Experiencing personal growth	U
		8. Get social support through self-help groups	U
		9. Managing and protecting	U
		10. Career exploration and development	U
		11. Teaching and training	U
		12. Adjusting expectations and having a positive outlook	U
		13. Seeking financial support	U
		14. Still anxious	U
		15. A vague hope	U
Travis and Juarez-Paz (27)	2	16. Struggling is the new normal	U
		17. The validated caregiver	U
		18. The isolated caregiver	U
Lee et al. (28)	5	19. The onset of tics shackles adolescents with TS	U
		20. The secular “me” from transmigration	U
		21. Peer recognition	U
		22. Opportunity for self-identity	U
		23. Adjustment to symptom-related situations	U
		24. Endeavoring to maintain the image of normalcy	U
Rindner (29)	5	25. Being caught ticcing	U
		26. Losing control over your tics in public	U
		27. Feeling different than others	U
		28. Being uneasy about disclosing your TS to others	U
		29 The intensity of embarrassment with TS decreases over time	U
		30. Use distraction	U
		31. Use of relaxation techniques	U
		32. Talk about your feelings with others	U
		33. Adopt normalizing behaviors	U
		34. Accept yourself	U
Ludlow et al. (30)	4	35. Coping with children’s challenging behavior	U
		36. Misconceptions and lack of understanding of professionals and the lay public	U
		37. Negative experiences of children’s education	U
		38. Support and services for families with TS	U
Edwards et al. (31)	4	39. Tic conceptualization	U
		40. Awareness of urges and tics	U
		41. Causes of tics	N
		42. Emotional impact	U
		43. Social impact	U
		44. Occupational (daily living) impact	U
		45 Physical impact	U
		46. Coping with TS	U
Cutler et al. (32)	4	47. Tics get in the way and are hard to manage	U
		48. TS is more than just tics	U
		49 Others not understanding involuntary behaviors	U
		50. Bullying and teasing	U
		51. Worrying about what others think	U
		52. Distracting and attention-consuming	U
		53. Tourette syndrome is one part of who I am	U
Lee et al. (33)	5	54. Uncontrollable body	U
		55. The loneliness of not being understood	U
		56. Interference with academic performance	U
		57. Compromising one’s self to integrate into society	U
		58. Conflict between autonomy and authority	U
		59. Helping factors in developing self-identity	U
		60. Two-faced	U
		61. The power of accepting that TS is a part of you	U

U, unequivocal; N, not supported.

Qualitative studies have been conducted as follows: two in the USA (27, 29), two in Taiwan (28, 33), two in the UK (30, 32), one in South Korea (26), and one in Canada (31).

This review included a total of 106 participants. Five studies (28, 29, 31–33) included children with TS. The children with TS included 54 males and 16 females. Three studies (26, 27, 30) included caregivers. The caregivers were five males and 31 females. Patients with TS ranged in age from six to 20 years.

### Quality of the included studies

All of the included studies met at least six of the ten criteria of the JBI Critical Appraisal Checklist for Qualitative Research (24): Lee et al. (28), Rindner (29), and Lee et al. (33) met all the criteria. Kim et al. (26) met nine criteria. Ludlow et al. (30), Edwards et al. (31), and Cutler et al. (32) met eight criteria and Travis et al. (27) met six criteria. Among the items, Q1 was met in three studies (28, 29, 33), Q4 in seven studies (26, 28–33), Q6 in four studies (26, 28, 29, 33), and Q7 in seven studies (26, 28–33). The remaining criteria were satisfied by all of the included studies. Overall, the methodological quality of the studies was good, because they met the majority of the predetermined criteria. No studies were excluded from the synthesis because they were all of good quality (Table 3).

**TABLE 3:** Quality assessment of the included studies.

Study	Q1	Q2	Q3	Q4	Q5	Q6	Q7	Q8	Q9	Q10
Kim and Tak (26)	U	Y	Y	Y	Y	Y	Y	Y	Y	Y
Travis and Juarez-Paz (27)	U	Y	Y	N	Y	N	N	Y	Y	Y
Lee et al. (28)	Y	Y	Y	Y	Y	Y	Y	Y	Y	Y
Rindner (29)	Y	Y	Y	Y	Y	Y	Y	Y	Y	Y
Ludlow et al. (30)	U	Y	Y	Y	Y	N	Y	Y	Y	Y
Edwards et al. (31)	U	Y	Y	Y	Y	N	Y	Y	Y	Y
Cutler et al. (32)	U	Y	Y	Y	Y	N	Y	Y	Y	Y
Lee et al. (33)	Y	Y	Y	Y	Y	Y	Y	Y	Y	Y
Total%	38	100	100	88	100	50	88	100	100	100

Y, yes; U, unclear; N, no; Joanna Briggs Institute (JBI) Critical Appraisal Checklist for Qualitative Research.

Q1, Is there congruity between the stated philosophical perspective and the research methodology?

Q2, Is there congruity between the research methodology and the research Question or objectives?

Q3, Is there congruity between the research methodology and the methods used to collect data?

Q4, Is there congruity between the research methodology and the representation and analysis of data?

Q5, Is there congruity between the research methodology and the interpretation of results?

Q6, Is there a statement locating the researcher culturally or theoretically?

Q7, Is the influence of the researcher on the research, and vice-versa, addressed?

Q8, Are participants, and their voices, adequately represented?

Q9, Is the research ethical according to current criteria or, for recent studies, is there evidence of ethical approval by an appropriate body?

Q10, Do the conclusions drawn in the research report flow from the analysis or interpretation of the data?

### Summary of the findings and ConQual assessment

Sixty-one findings were extracted from eight studies. Sixty findings were considered unequivocal. One finding was unsupported and was excluded. After the exclusion of the unsupported findings, 60 extracted findings were aggregated into 15 categories. The four synthesized findings were derived from 15 categories: (i) continuation of challenging daily life, (ii) denying that TS causes emotional distress, (iii) accepting and understanding TS as part of oneself, and (iv) looking to the future. The illustrative quotations are presented in Supplementary Table 2.

All synthesized findings received high ConQual scores (Table 4).

**TABLE 4:** Summary of the findings and ConQual assessment.

Synthesized findings	Type of research	Dependability	Credibility	ConQual score	Comments
Continuation of challenging daily life	Qualitative	High (no change)	High (no change)	High	Dependability unchanged: Of the seven primary studies, three studies addressed all five dependability questions, three studies addressed four dependability questions, and one study addressed two dependability questions. Credibility unchanged: 18 findings were included; all were unequivocal
Denying that TS causes emotional distress	Qualitative	High (no change)	High (no change)	High	Dependability unchanged: Of the six primary studies, four studies addressed all five dependability questions, and two studies addressed four dependability questions. Credibility unchanged: 20 findings were included; all were unequivocal
Accepting and understanding TS as part of oneself	Qualitative	High (no change)	High (no change)	High	Dependability unchanged: Of the seven primary studies, four studies addressed all five dependability questions, two studies addressed four dependability questions, and one study addressed two dependability questions. Credibility unchanged: 17 findings were included; all were unequivocal
Looking to the future	Qualitative	High (no change)	High (no change)	High	Dependability unchanged: Of the three primary studies, three studies addressed all five dependability questions. Credibility unchanged: five findings were included; all were unequivocal

### Synthesized finding 1: Continuation of challenging daily life

**FIGURE 2:**
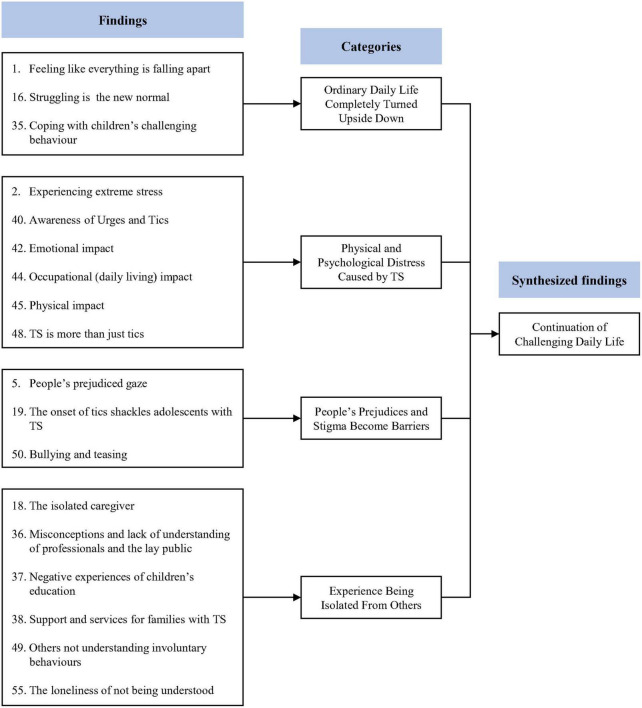
Synthesized finding 1: Continuation of challenging daily life.

This synthesis was based on 18 findings, aggregated into four categories (Figure 2). Children with TS and their caregivers reported experiencing radical changes in their daily lives. They also reported physical and psychological distress due to TS. At the same time, they reported that other people’s prejudices and stigma about TS trap them and that people’s ignorance of TS isolates them.

#### Category 1: Ordinary daily life completely turned upside down

Tourette’s syndrome had negative consequences in several areas of daily life (26, 30). It is often difficult for caregivers of children with TS to lead normal daily lives (26, 30). Children’s uncontrollable TS symptoms can instill feelings of frustration and guilt in their caregivers (26, 27, 30). The arduous life of fighting and struggling with symptoms becomes routine for children with TS and their caregivers (27).

#### Category 2: Physical and psychological distress caused by Tourette’s syndrome

Children with TS and their caregivers report various physical and psychological distress caused by TD (26, 31, 32). Children with TS reported physical distress such as nosebleeds due to TS, as well as premonitory urges such as itchiness and tingling (31). In addition, children with TS reported psychological difficulties, such as difficulty concentrating, embarrassment, and anger due to symptoms (31, 32). Meanwhile, caregivers also reported that whenever their children showed symptoms of TS that were difficult to control, they found it painful to watch them and sometimes had suicidal thoughts (26).

#### Category 3: People’s prejudices and stigma become barriers

Prejudice and stigma can become barriers for children with TS (26, 28, 32). Children with TS were found to be disadvantaged and underestimated, simply because they had TS (26, 28). They were reported to often become the target of bullying because they were different from others (32).

#### Category 4: Experience of being isolated from others

Children with TS and their caregivers experience feelings of isolation due to general public indifference and ignorance of disability (27, 30, 32, 33). Children with TS were often misunderstood or came into conflict with others because their behavior was different from that of people without TS, and they felt lonely as a result (32, 33). Their caregivers also reported feeling isolated and alienated due to a lack of support and the fact that their children’s symptoms were not understood by others (27, 30).

### Synthesized finding 2: Denying that Tourette’s syndrome causes emotional distress

**FIGURE 3:**
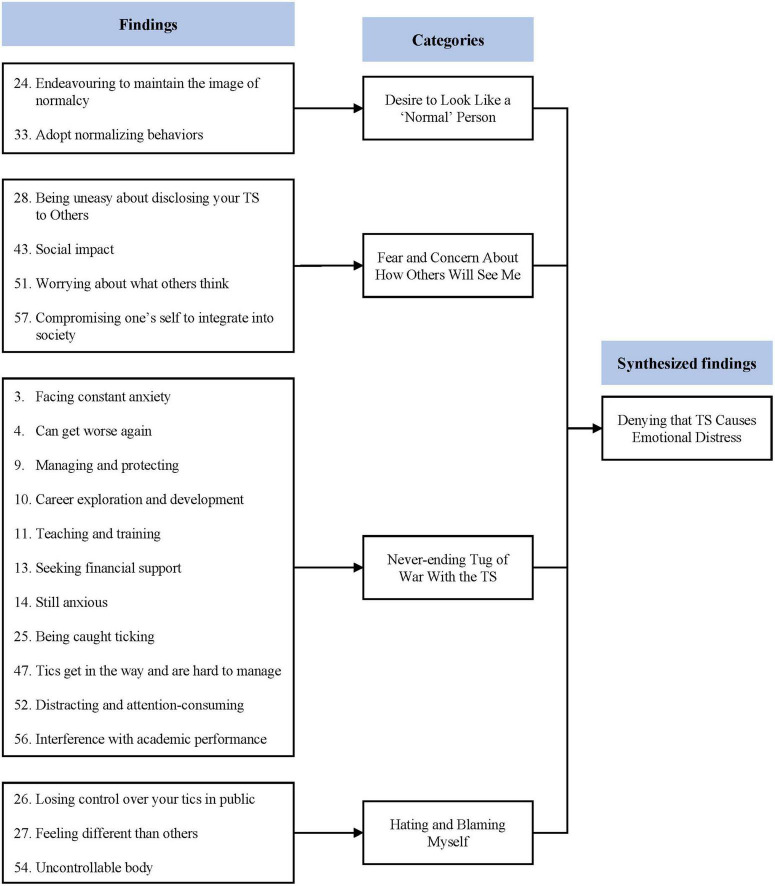
Synthesized finding 2: Denying that TS causes emotional distress.

This synthesis was based on 20 findings, aggregated into four categories (Figure 3). Children with TS suppressed their tics because they wanted to resemble their typically developing peers. However, this attitude maintained and exacerbated their emotional distress. Thinking that they were different from others made them worry about others’ views. Indeed, when the children lost control of their TS, they blamed themselves and felt guilty.

#### Category 1: Desire to look like a “normal” person

Children with TS have been reported to try and pretend to be “normal” (28, 29). They have been reported to encourage themselves to think and act like “normal” people (29) and mentioned that they wanted to be treated on an equal basis to people without TS. They even denied some of their rights, such as special examination room, fearing they might look different from others (28).

#### Category 2: Fear and concern about how others will see me

Children with TS can experience ambivalent social interactions (31). For example, some people accept TS, whereas others try to control it. They are sometimes bullied. Because of this ambivalence, children with TS are constantly trying to read others’ minds (32, 33). Even disclosing one’s TS to a close friend is usually done after careful consideration (29, 33).

#### Category 3: Never-ending tug of war with the Tourette’s syndrome

Children with TS and their caregivers constantly struggle to control the symptoms of TS (26, 29, 32, 33). They have been reported to have difficulties in school and daily life because they had to keep suppressing their TS from exposure (29, 32, 33). Paradoxically, this stress causes TS symptoms, creating a vicious cycle (32). Meanwhile, caregivers of children with TS reported being constantly worried and anxious about the future of their child (26).

#### Category 4: Hating and blaming myself

Children with TS tried to control their symptoms. However, it is difficult to completely suppress them. They blamed themselves and felt guilty whenever they lost control of their TS (29, 33). They even called themselves *freaks* or *werewolves* and perceived themselves as alien beings that could not assimilate with those around them (29, 33).

### Synthesized finding 3: Accepting and understanding Tourette’s syndrome as part of oneself

**FIGURE 4:**
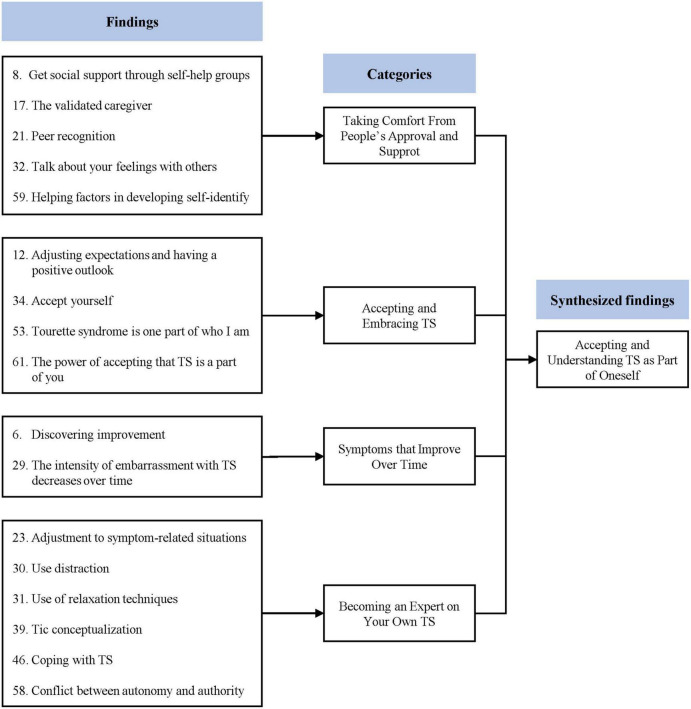
Synthesized finding 3: Accepting and understanding TS as part of oneself.

This synthesis was based on 17 findings, aggregated into four categories (Figure 4). Some children with TS and their caregivers came to accept and understand TS. The recognition and support they received from others became the driving force for them to live with TS. Over time, the symptoms of TS and distress caused by TS symptoms were reported to decrease. At the same time, the children learned more about TS and themselves.

#### Category 1: Taking comfort from people’s approval and support

Children with TS and their caregivers have been reported to find comfort in receiving support from others (26–29, 33). Parents were able to receive practical help in raising children with TS through self-help groups, and by receiving other people’s recognition of their efforts, they were able to relieve some of their guilt about their children (26, 27). Children with TS can receive emotional support from family, friends, and experts, and, based on these experiences, they can learn to accept themselves and gain confidence (28, 29, 33).

#### Category 2: Accepting and embracing Tourette’s syndrome

Children with TS and their caregivers can come to acknowledge and accept TS (26, 29, 32, 33). Parents that lowered their expectations of their children were able to see their children and TS as they really are (26). Meanwhile, children with TS can come to acknowledge that TS is a part of them and their personality (29, 32, 33). They no longer try to avoid or suppress their condition (29).

#### Category 3: Symptoms that improve over time

Tourette’s syndrome symptoms and secondary distress caused by TS tend to decrease over time (26, 29). Children with TS reported that the embarrassment caused by TS decreased as they grew (29). Parents reported feeling that as their children’s symptoms improved, their behavior changed (26).

#### Category 4: Becoming an expert on your own Tourette’s syndrome

Children with TS can come to understand more about TS and themselves (28, 29, 31, 33). Initially, children with TS tend to recognize TS as a simple habit, but gradually can come to recognize it as a disorder and understand the characteristics of TS (31). Then, they can learn how to deal with TS on their own, and as they understand the situation and context in which TD appears, they can become experts on their own TS (28, 29, 31, 33).

### Synthesized finding 4: Looking to the future

**FIGURE 5:**
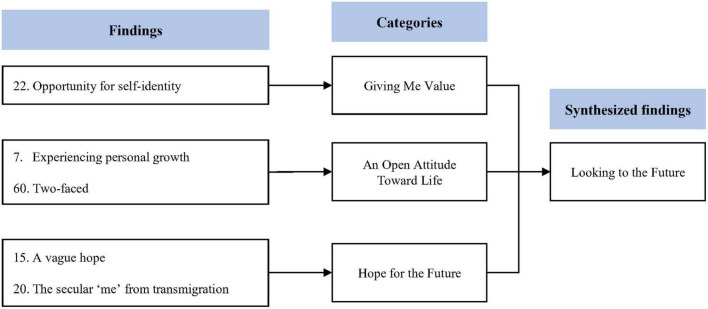
Synthesized finding 4: Looking to the future.

This synthesis was based on five findings aggregated into three categories (Figure 5). TS is a painful experience for both patients and their caregivers. However, some children reported personal growth in relation to their TS. Children with TS were able to recognize their own values. Furthermore, patients and their caregivers can learn to have a more open attitude toward life and be more optimistic toward the future.

#### Category 1: Giving me value

Children with TS were able to recognize their own values (28). They can learn to not only develop their own strengths and abilities but also to love themselves before seeking approval from others (28).

#### Category 2: An open attitude toward life

Children with TS and their caregivers have also reported positive changes due to TS (26, 33). They reported that due to TS, they had a more empathetic attitude when dealing with people in difficult situations (26, 33). Some children and their caregivers reported that they came to understand that TS is not bad and can lead to personal growth (33).

#### Category 3: Hope for the future

Children with TS and their caregivers can learn to be more optimistic about their future (26, 28). As children with TS mature, they often become more confident and are no longer as worried about the perception of others (28). They can become more optimistic about the future and experience personal growth. Similarly, caregivers have been reported to have more hope for the future as their children gradually adjust to living with TS (26).

## Discussion

### Principal findings

In this review, we aggregated qualitative evidence on the experiences and perspectives of children with tic disorder or TS and their caregivers and developed four synthesized findings: (i) continuation of challenging daily life, (ii) denying that TS causes emotional distress, (iii) accepting and understanding TS as part of oneself, and (iv) looking to the future. These syntheses summarized the pain that children with TS and their caregivers experience and how they live with the symptoms.

The first synthesized finding, “continuation of challenging daily life,” covers the various problems experienced by children with TS and their caregivers in daily life. TS is major stressor that impact daily routines. Because of tics, daily life can be challenging, with children and their caregivers experiencing hardships and adversity. Children with TS report physical pain, such as nosebleeds, and various premonitory urges, such as itchiness and tingling. In addition, these TS symptoms interfere with daily life and cause negative emotions, such as embarrassment, worry, and anger. These problems are related to difficulties in achieving adequate academic achievement in children with TS (20), and 76% of patients with TS experience depression-related symptoms (6).

Social deprivation is another problem that is frequently faced by children with TS and their caregivers. They often have to tolerate the prejudices of others, and children with TS are bullied simply because they have a disability. In addition, others’ ignorance of and indifference to TS are major causes of their feelings of alienation. Several anti-stigma programs have been launched in the past to reduce prejudice against mental disorders, and these have led to active public education that mental disorders, like other diseases, have biological causes (34). Consequently, the understanding of the biological causes of mental disorders has deepened since the 1990s, and the need for specialists to treat mental disorders has been recognized (35). However, despite these efforts, prejudice and stigma toward people with mental disorders did not decrease but instead worsened (35). Therefore, prejudice and stigma against mental disorders remain prominent issues to be resolved.

The second synthesized finding, “denying that TS causes emotional distress,” means that avoidant and repressive coping strategies for TS cause secondary pain. Children with TS have a strong desire to appear “normal” to others and often use avoidant and repressive strategies to hide their TS symptoms. However, this can also lead to chronic stress. This stress leads to more severe TS, which ultimately leads to a paradoxical situation in which attempts to eliminate symptoms aggravate them. The pain experienced by patients with TS is due to the ironic process of thought inhibition (36), suggesting that avoidant and repressive coping strategies may be adaptive in the short term but do not provide much help in eventual problem-solving (37). The problem, in which attempts to suppress or mask symptoms have the opposite effect, decreasing mental health, has also been discussed in the case of autism (38, 39). Recent studies found that camouflaging autism negatively affects the psychological wellbeing of people with autism (39), and acceptance of autism related behaviors was more effective in stigma reduction than mere disclosure of an autism label to peers (40). Similarly, suppressing tics is an aspect of camouflaging, which risks psychological wellbeing, such as loss of self-esteem and emotional problems. These problems are experienced as chronic stress that causes more severe TS in children, eventually leading to the paradoxical situation in which suppressing tics increases tics.

In addition, this finding revealed that when these avoidant coping strategies for TS fail, children with TS show negative thinking patterns that ruminate in the past, such as self-blame or guilt. Rumination is a dysfunctional thinking pattern that repeatedly and passively recalls the causes and effects of distress, even though it is not helpful in problem solving (41). Considering that rumination increases depression and negative thoughts and impairs problem- solving abilities (37), self-blame may have a negative effect on the overall cognitive, emotional, and behavioral aspects of children with TS. Furthermore, rumination delays adaptive coping strategies and increases avoidant coping strategies (37, 42). Self-blame is a key factor that increases the avoidance and suppression of symptoms in children with TS, which can lead to a vicious cycle.

The third synthesized finding, “accepting and understanding TS as part of oneself,” suggests effective strategies that children with TS can use to cope with their symptoms. Children with TS can seek social support to reduce symptom distress, and they can acknowledge and accept TS as a part of themselves. These coping strategies are effective emotion regulation methods and can be a personal strength and source of growth (43, 44). Post-traumatic stress disorder (PTSD) is a pathological consequence of a traumatic event, and post-traumatic growth (PTG) is a state in which the effect of a traumatic event has a positive outcome. A key factor that induces PTG is intentional cognitive processing, which attempts to integrate the traumatic event with the individual’s representation (44). To promote such intentional rumination, it is important to control intense and negative emotions related to trauma, and emotion regulation strategies such as seeking social support and mindfulness exercises can be effective alternatives (43, 44). Children with TS and their caregivers reported fewer TS symptoms and distress over time. Since the symptoms of tics tend to decrease after adolescence (3) and the ways of coping with tics have changed over time, it could be expected that secondary distress caused by tics, such as embarrassment or worry, has decreased. Indeed, the positive prognosis of TS is confirmed in recent research (45) and is hypothesized to be related to better acceptance of tics by the environment (46). Children with TS can gradually become experts on TS and themselves if they confront and accept their symptoms rather than try to suppress them. This is thought to be similar to the process of moving from PTSD to PTG through intentional rumination during stressful events.

The fourth synthesized finding, “looking to the future,” refers to a change in the autobiographical narrative about the symptoms. Specifically, this finding suggests that individuals can gain from a disability. As children with TS overcome the hardships of stigma related to tics, they can recognize their own values. This opens up the opportunity of personal growth including discovering the positive aspects of TS identity. These changes are similar to PTG, which is represented by personal value perceptions, openness, appreciation for life, positive interpersonal relationships, and a more existential and spiritual life (44). The idea that hardship and adversity can be the basis for growth has been known for a long time. For example, according to the teachings of Hinduism, Buddhism, and Islam, as well as the early ideas and writings of ancient Hebrews, Greeks, and Christians, hardships can be a force for growth (47). TS is a difficult challenge for both children and their caregivers. However, the findings of this study show that children with TS and their caregivers can lead a better life despite the hardships and adversity they face.

### Limitations

This study had several limitations.

First, this study may be limited in that it included the stories of children with TS and their caregivers in a qualitative systematic review. This is because the number of individual studies was not sufficiently large to conduct a qualitative systematic review by dividing the subjects into groups. Nevertheless, synthesizing patients’ and caregivers’ stories together can help gain a comprehensive understanding of TS.

Second, there may be a problem in the quality of the research because this review includes one thesis (29) that has not undergone peer review. However, the thesis was selected after undergoing a quality evaluation.

Third, this review did not exclude a specific language in the selection of studies but limited the selection of databases due to language limitations. For example, although we included a Korean database, we did not include a Chinese or Japanese database.

### Clinical implications

This study synthesizes the experiences and perspectives of children with tic disorder or TS and their caregivers and suggests clinical implications.

First, according to these findings, social deprivation, such as prejudice or bullying from others and indifference to TS, is one of the challenges experienced by children with TS and their parents. Social support can be a driving force for children and their parents to live a life with confidence and less guilt. In other words, the prognosis of disability is expected to depend on the type and quality of the social experience. Indeed, bullying by peers could be one of the causes of worsening symptoms in patients with TS (45). Therefore, appropriate social support should be provided to children with TS and their caregivers in the clinical setting. The provision of a professional group that can promote social support could be helpful. Furthermore, efforts to change the public perception of disability, such as education about TS and the stigma effect, may reduce the risk of social deprivation experienced by children with TS and their caregivers.

Next, according to the findings, it was found that the attitude of avoiding or suppressing TS increases the secondary distress caused by symptoms. Moreover, if avoidant and suppressive coping strategies fail, there is a risk of exacerbating self-blame. In contrast, accepting TS, unlike suppression or avoidance, can function as an effective emotion regulation strategy (48, 49), and this ability to regulate emotion helps one to understand oneself and their condition systematically. In other words, it could be expected that the prognosis of disability depends on both attitudes toward TS and the coping strategies used. Therefore, it is necessary to develop effective emotion regulation strategies for children with TS in a clinical setting. Specifically, it is necessary to cultivate a non-judgmental attitude toward TS symptoms and oneself. Mindfulness-based treatments (e.g., mindfulness-based stress reduction, acceptance and commitment therapy, and dialectical behavioral therapy) should be considered.

Acceptance from others is an important factor in predicting a positive prognosis for TS (45, 46). Therefore, acceptance of TS should be performed in social contexts beyond individual patients (12). First, it is possible to introduce acceptance-based education to reduce the stigma effect on mental disorders for caregivers. Afterward, it is necessary to gradually expand the target of such education to teachers, peers, and local community residents. Standardized interventions to prevent stigma must be developed to implement such a wide range of education and policy improvements must be made to support it.

## Conclusion

This review synthesizes the experiences and perspectives of children with tic disorder or TS and their caregivers. Children with TS and their caregivers reported various physical and psychological distress caused by TS and also experienced social deprivation, which was aggravated through the denial or suppression of TS. However, having to overcome adversity and hardships associated with TS provides children with an opportunity for personal growth, including discovering positive aspects of TS identity. We anticipate that this growth may have been possible because, with appropriate social support, they were able to accept TS as a part of themselves. Therefore, clinicians in the clinical setting need to provide appropriate social support to children with TS and their caregivers as well as implement specialized treatments that allow them to accept their disabilities.

## Data availability statement

The original contributions presented in this study are included in the article/Supplementary material, further inquiries can be directed to the corresponding author.

## Author contributions

H-WS, S-IY, SH, HL, and ML selected the studies. H-WS, S-IY, and ML extracted the data. S-IY aggregated the findings according to similarity in meaning, merged them into categories, and wrote the first draft of the manuscript. H-WS then reviewed the results. H-WS, JWK, and S-YC critically revised the manuscript. All authors read and approved the final manuscript.
