# Supplementary material for: How do children with Tourette’s syndrome and their caregivers live with the disorder? A systematic review of qualitative evidence
Source: Front Psychiatry. 2022 Sep 29;13:992905. doi: 10.3389/fpsyt.2022.992905 (PMC9557735; doi:10.3389/fpsyt.2022.992905)
Supplement: Supplementary file 1 [file Table_1.DOCX]

Supplementary Table 1. Search strategies for each database

|  | **Search strategies** |
| --- | --- |
| PubMed | #1. Tics [MH]  #2. Tic Disorders [MH]  #3. Tourette Syndrome [MH]  #4. tic or tics or Tourette* [Title/Abstract]  #5. #1 OR #2 OR #3 OR #4  #6. Qualitative Research [MH]  #7. Interviews as Topic [MH]  #8. (qualitative or themes) [Title/Abstract]  #9. (focus-group* OR ethnograph* OR phenomenology OR grounded-theory) [Title/Abstract]  #10. #6 OR #7 OR #8 OR #9  #11. #5 AND #10 |
| EMBASE | #1. ‘tic’/exp  #2. ‘gilles de la tourette syndrome’/exp  #3. (tic OR tics OR tourette$):ab,ti  #4. #1 OR #2 OR #3  #5. ‘qualitative research’/exp  #6. ‘interview’/exp  #7. (qualitative OR themes):ti,ab  #8. (focus-group* OR ethnograph* OR phenomenology OR grounded-theory):ti,ab  #9. #5 OR #6 OR #7 OR #8  #10. #4 AND #9 |
| CINAHL | #1. (MH “tic+”)  #2. (MH “Tourette Syndrome”)  #3. TI(tic OR tics OR Tourette) OR AB(tic OR tics OR tourette)  #4. #1 OR #2 OR #3  #5. (MH “Qualitative Studies+”)  #6. (MH “Interviews+”)  #7. TI(qualitative OR themes) OR AB(qualitative OR themes)  #8. TI(focus-group* OR ethnograph* OR phenomenology OR grounded-theory) OR AB(focus-group* OR ethnograph* OR phenomenology OR grounded-theory)  #9. #5 OR #6 OR #7 OR #8  #10. #4 AND #9 |
| PsycARTICLES | #1. MAINSUBJECT.EXPLODE(“Tics”)  #2. MAINSUBJECT.EXPLODE(“Tourette Syndrome”)  #3. TI(tic OR tics OR tourette) OR AB(tic OR tics OR tourette)  #4. #1 OR #2 OR #3  #5. MAINSUBJECT.EXPLODE(“Qualitative Methods”)  #6. MAINSUBJECT.EXPLODE(“Interviews”)  #7. TI(qualitative OR themes) OR AB(qualitative OR themes)  #8. TI(focus-group* OR ethnograph* OR phenomenology OR grounded-theory) OR AB(focus-group* OR ethnograph* OR phenomenology OR grounded-theory)  #9. #5 OR #6 OR #7 OR #8  #10. #4 AND #9 |
| KMbase | #1. (((([ALL=틱장애] OR [ALL=틱 장애]) OR [ALL=뚜렛]) OR [ALL=tic disorder]) OR [ALL=tourette])  #2. ((((([ALL=질적] OR [ALL=포커스 그룹]) OR [ALL=문화기술지]) OR [ALL=현상학]) OR [ALL=근거이론]) OR [ALL=qualitative])  #3. #1 AND #2 |
| SienceON | #1. ("틱장애"\|"틱 장애"\|"뚜렛"\|"tic disorder"\|"tourette")  #2. ("질적"\|"포커스 그룹"\|"문화기술지"\|"현상학"\|"근거이론"\|"qualitative")  #3. #1 AND #2 |
| RISS | #1. 전체 : 틱장애 <OR> 전체 : 뚜렛 <OR> 전체 : tic disorder <OR> 전체 : tourette <AND> 전체 : 질적  #2. 전체 : 틱장애 <OR> 전체 : 뚜렛 <OR> 전체 : tic disorder <OR> 전체 : tourette <AND> 전체 : 포커스 그룹  #3. 전체 : 틱장애 <OR> 전체 : 뚜렛 <OR> 전체 : tic disorder <OR> 전체 : tourette <AND> 전체 : 문화기술지  #4. 전체 : 틱장애 <OR> 전체 : 뚜렛 <OR> 전체 : tic disorder <OR> 전체 : tourette <AND> 전체 : 현상학  #5. 전체 : 틱장애 <OR> 전체 : 뚜렛 <OR> 전체 : tic disorder <OR> 전체 : tourette <AND> 전체 : 근거이론  #6. 전체 : 틱장애 <OR> 전체 : 뚜렛 <OR> 전체 : tic disorder <OR> 전체 : tourette <AND> 전체 : qualitative  #7. #1 OR #2 OR #3 OR #4 OR #5 OR #6 (After removing duplicates) |

CINAHL=Cumulative Index to Nursing and Allied Health Literature; KMbase=Korean Medical Database; RISS=Research Information Sharing Service
