# Supplementary material for: How do children with Tourette’s syndrome and their caregivers live with the disorder? A systematic review of qualitative evidence
Source: Front Psychiatry. 2022 Sep 29;13:992905. doi: 10.3389/fpsyt.2022.992905 (PMC9557735; doi:10.3389/fpsyt.2022.992905)
Supplement: Supplementary file 2 [file Table_2.DOCX]

Supplementary Table 2. Illustrative quotations for each finding

| **Study** | **Numbered findings** | **Quotations** |
| --- | --- | --- |
| Kim and Tak (22) | 1. Feeling like everything is falling apart | "나는 애를 낳아서 어떻게 키울 것인가 그런 상상들을 많이 하잖아요. 나도 애를 낳아서 이제 딸이니까 이쁘게 키우고 싶었는데 애가 병이 딱 생기면서부터 인생이란 게 원래 계획대로 되는 게 아무것도 없지만 모든 게 무너진 거에요. 좀 이쁘고 그렇게 키우고 싶은데 약을 먹으면서 애가 살이 찌고 막 이렇게 되면서. 내가 가지고 있던 생각들이 다 한꺼번에 무너지고. 누구를 만나면 내가 어느 정도고, 남편이 사회적으로 위치가 있고, 그런 게 어느 정도 갖춰지던 시기였는데 (딸이) 틱이 딱 생기면서부터 그게 다 무너졌어요. 그래서 모든 자존심 그런 거를 저는 다 내려놨어요." (P8, p.165) |
|  | 2. Experiencing extreme stress | "저는 참다 참다 안 되니까 그냥 죽어야 되겠다, 이런 생각이 순간 확 들었던 것 같아요. (…) 생각나는 게 정말 죽음밖에 없고, '스트레스를 해소해야지, 뭐 운동을 해서 해소해야지' 이런 생각까지 갈 상황이 아닌 것 같아요." (P3, p.166) |
|  | 3. Facing constant anxiety | "점점 성인이 돼서 얘가 사회생활을 좀 잘 했으면 하는 그런 단계들이 있잖아요. 고등학교 때는 졸업을 하고 대학 문제라든가, 또 대학을 들어가면 졸업을 하고 직장 문제, 이런 것들이 끝이 없이 있잖아요. 평범한 아이 키우는 사람들도 마찬가지이긴 하겠지만, 그게 뚜렛하고 연관돼서 더 걱정이 되는 거죠. 얘가 폭력적인 성향이 있기 때문에 다른 사람하고 트러블이 생겼을 때 그런 거를 조절하지 못하고, 다툼이 되지 않을까 막 그런 걱정들이 계속 오는 거죠." (P3, p.166) |
|  | 4. Can get worse again | "옛날에 심할 때 100이었으면 지금은 한 10밖에 안되잖아요. 그런데 혹시 또 누가 뭐라 해서 그런 것들이 ㅇㅇ이에게 나타날까 봐. ㅇㅇ이 같은 애들이 감정 기복이 심하니까 또 그러면 전화해서 엄마!! 막 그럴까 봐 그게 항상 조마조마 한 거죠." (P8, p.167) |
|  | 5. People’s prejudiced gaze | "아무리 미디어나 이런 데서 틱과 뚜렛에 대해서 알려준다 하더라고 거의 대부분 사람들이 아직은 모르는 증상이어서 아무 전제조건, 사전지식 없이도 아이가 안 좋게 보여지는 거는 있지요. (…) 버스 타고 있다가 애가 틱을 하면 다 돌아보거든요. 그런 면에서 아직도 아이가 편안해지기는 멀었다는 뜻이고 틱이 없다고 가정했을 때 다른 사람이 아이를 보는 관점이 지금하고는 다를 텐데 지금은 아무 잘못도 없이 점수를 마이너스를 받고 시작을 한다는 생각을 해요." (P4, p.168) |
|  | 6. Discovering improvement | "어떨 때는 몇 번 참다가 "나 지금 엄청 참고 있는 거다, 예전 같으면 이미 엄마한테 벌써 소리 질렀고, 나쁜 말 했고, 이렇게 상(을 탁) 쳤는데, 지금 내가 꾹꾹 참고 있는 거다"라고 부들 부들 부들 이렇게 하면서 내가 지금 참고 있으니가 더 이상 나한테 공격하지 말고 내 얘기 좀 들으시라고 하는 거예요. 옛날 같으면 이미 나한테 폭발을 했겠죠. (이런 부분이 예전과 달리) 나아진 부분이에요." (P1, p.169) |
|  | 7. Experiencing personal growth | "가족 사이의 관계도 그렇고 어디 가서든 (…) 다른 사람들 보는 시각도 다 달라지고. 만약에 우리가 틱이 없었다면 다른 사람을 배려하거나 사람을 (차별하지 말고) 똑같이 봐야 된다라는 생각은 늦게 가졌을 것 같아요." (P9, p.169) |
|  | 8. Get social support through self-help groups | "모임이 없고 이런 거(자조모임에 참석)하는 사람을 못 만나고 혼자 있었다면 지금 어떻게 됐을지 상상이 안 가요. 같은 틱을 하는 친구들을, 엄마들을 만나서 얘기도 하고 그들이 어떻게 하고 있는지 듣기도 하고. 병원 가는 거 하고는 다른 것 같아요. (…) 약 이름도 알고 어떻게 하면 좋다라는 것도 알고 가족이 어떻게 해야 된다, 이런 대처방법들을 알아가는 게 많이 도움이 됐죠." (P9, p.170) |
|  | 9. Managing and protecting | "항상 불안해서 (아이가) 뭘 하고 있는지 알아야 제가 대처를 할 수 있다는 생각이 있는 것 같아요. 저녁에 들어오는 것도 항상 체크하고 얘도 내가 그걸 신경쓰고 있다는 걸 알아요." (P9, p.170) |
|  | 10. Career exploration and development | "최근 들어 그나마 발견한 게 요리하고 먹는 거하고 관련이 된 거. (…) 한 이십 년만에 아이한테 '아 얘가 이거는 괜찮다, 남들보다 뛰어나다' 하는 걸 찾았어요." (P4, p.171) |
|  | 11. Teaching and training | "내가 세상 사는 이런 것들을 가르쳐. 달라는 대로 다 주고 (그러면) 사기를 당하기도 쉬운 거야. (아이가 )세상 물정을 모르니까." (P6, p.171) |
|  | 12. Adjusting expectations and having a positive outlook | "목표치가 아이에 대해서도 그렇고 남편에 대해서도 낮아진 거. (웃음) 눈이 낮아진 거죠. (웃음) 얘가 막 요만큼만 해도 그게 기쁘고 요만큼만 변화되어도 행복하다. 그런 게 생긴 것 같아요." (P3, p.171) |
|  | 13. Seeking financial support | "지금 사는 집이 애기 아빠 이름이지만 은연 중에 ㅇㅇ이를 줘서 먹고 살게 해야되지 않을까 생각은 있어요. 딸에게는 미안하지만 (…) 이 집은 ㅇㅇ이를 줘서 ㅇㅇ이가 평생 우리가 간 뒤에 먹고 살게끔 해줘야 되지 않을까 그런 생각이 있고 물론 경제적으로 독립하지 않아서 (계속) 뒷바라지 해줄 수 있으면 좋겠죠. 부모가 경제적인 걸 도움을 주어야 되지 않을까." (P9, p.172) |
|  | 14. Still anxious | 쟤가 저러고 있으면 안되는데 (싶어서) 답답하고, 일단은 아프니까 그렇다고는 하지만 그래도 활동을 해야죠. (…) (아이가) 밥 먹는 모습만 봐도 답답하고 그래서 제가 스트레스 때문에 다른 거 하기가 싫어지더라고요. (P9, p.172) |
|  | 15. A vague hope | "고등학교 때까지는 (다른 자녀와) 어마어마하게 달랐고 막연하게 가망이 없고 희망이 없어 너무 힘들어서 '과연 얘가 어떻게 (될까)?'라는 느낌이 너무 강했고, 지금은 (다른 자녀와) 많이 다르진 않아요. 그래도 둘째(다른 자녀)보단 어려움이 있을 수 있다는 생각은 드는데. 성실하고 규칙을 지키는 그런 면들이, 진심은 통한다고, 일하는 데 도움이 되지 않을까. (…) 고등학교 때까지는 막연한 깊은 절망이었고 지금은 막연한 희망." (P1, p.173) |
| Travis and Juarez-Paz (23) | 16. Struggling is the new normal | “It’s been hard. Oh my gosh . . . it’s been rocky because . . . we were struggling . . . it’s not easy.” (p.1483)  “he’s a difficult kid to parent . . . the days can be incredibly challenging . . . the hours can be challenging . . . there’s guilt that goes with . . . thinking about how is this going to make my life more difficult.” (p.1484)  “that’s when we really started our journey to really understand what TS was more about.” (p.1484)  “it’s exhausting to kind of have to feel like you are always having to explain where you are.” (p.1484) |
|  | 17. The validated caregiver | Another mother explained that hearing she was “doing a great job” was reassurance that TS “isn’t anything that (she) created” and “(I’ve) helped him deal with it better.” (p.1485)  They explained that supportive communication that validates their experiences is helpful (Authors, p.1487) |
|  | 18. The isolated caregiver | Another caregiver said that she wanted others to know that the behaviors of an individual with TS “cannot be controlled even if the individual looks like they are in control.” She also explained, "We actually do need to have a special parenting skill and no that doesn’t mean we are lacking in other parenting skills. Suggesting that my son’s behavior might be corrected by more discipline isn’t helpful at all. We can’t discipline a disability out of these children." (p.1486)  She said that the lack of understanding expressed by friends and family makes her feel “like (she) is in her own little world” which is “really challenging.” (p.1487)  Supportive communication that questions their experiences is isolating (Authors, p.1487) |
| Lee et al. (24) | 19. The onset of tics shackles adolescents with TS | "I cannot control my body at all. I just keep trembling and may even yell like a dog. I cry and yell at night until all of my family comes to see what has happened. The reason I cry is that I cannot tolerate the tics anymore. However, no one really understands me; otherwise they would not complain about the noise I make." (Participant K, p.466)  "My classmates look at me in a strange way, and they even impose discrimination on me. They wonder why they are different from me because I will show some strange actions. I feel that I am not a part of the peer group, and I suffer from discrimination." (Participant  A, p.467) |
|  | 20. The secular 'me' from transmigration | "The maturity of my senior high school classmates is one of the reasons why I have become more optimistic. However, I think the main reason is that I’ve also grown up, and my attitude has gradually been corrected. I do not regard myself as a freak now." (Participant  E, p.467) |
|  | 21. Peer recognition | "My classmates make me feel happy. Sometimes, we play so happily that I forget that I suffer from tics. I feel that I am a normal person and my disease has vanished. Therefore, my friends are really important to me." (Participant G, p.467) |
|  | 22. Opportunity for self-identity | I feel that I should try my best to accept myself without regarding myself as a disadvantaged person. Because I suffer from TS, I should develop more personal characteristics and strengths to cover up the disadvantage related to suffering from TS. I have to like  myself and identify with myself first, before winning recognition from my peers. (Participant J, p.467) |
|  | 23. Adjustment to symptom-related situations | "I am nervous about the onset of tics during school openings and examinations. I will start to scream out when I am nervous. I think pressure may aggravate the onset. However, as long as the pressure is alleviated, the symptoms are not severe." (Participant G, p.468)  "I don’t want to make odd movements or sounds. My discomfort becomes a joke in my class and my classmates tease me about it. I sure feel sad and humiliated. Sometimes, I pretended I don’t hear the gossip, and then after school, I listened to music to obtain relief or play basketball to forget the sadness." (Participant K, p.468) |
|  | 24. Endeavouring to maintain the image of normalcy | Although I can play happily with my friends and earnestly study with them, I still intend to suppress my tics because I don’t want to be seen as different to my friends. (Participant L, p.468) |
| Rindner (25) | 25. Being caught ticcing | "Um, Well, Well not really. I mean sometimes it - I was - I was embarrassed when there was a boy that I was sitting over in front of. He's my other enemy. And, he didn’t know I had tourette's or anything, and we were sitting in assembly and I did a tic and he said "Don't do that!" And i said, "i can't help it!" And so I turned around and I did it again and he said, "Stop!" And I couldn't help it-I just coudn't stop because I was in assembly and those makes me tic a lot, And he said "STOP doing that!" and I said, "I can't help it!" and he said, "Yes you can! Quit that!" And I felt very hurt and embarrassed." (p. 95) |
|  | 26. Losing control over your tics in public | "I did usually because I hate for them (the tics) to come… I (sic) just be like you're coming out at the wrong time, just go away and come back later. But I did get embarrassed because they would just be outrageous (meaning the severity of the tics)" (p. 97) |
|  | 27. Feeling different than others | "I felt like someone who had been changed into a werewolf" (p. 98) |
|  | 28. Being uneasy about disclosing your TS to others | "It (whether I would tell someone about my TS) depends on who I'm talking to. If I'm talking to someone who is really fast and they wouldn’t care or the would cut you off when you try to tell them. I just leave it alone and not tell them. But if somebody was really anxious to know what is it, and they couldn't and they seem like they're a person who could understand it, then I would really let out to them" (p. 100) |
|  | 29. The intensity of embarrassment with TS decreases over time | "Yeah probably a 10. Even a 10 to an 8… Like I had a tic, It's because of my tongue biting. I started biting my tongue, so I had to drink water so I like could rinse my mouth out and cool my tongue down. (생략) (L.S., 9/24/03, p. 103)  "I'm not embarrassed at all. I really aren't, I mean, err, It's like zero right now" (L.S., 9/24/03, p. 104) |
|  | 30. Use distraction | "I'm just like you know, I talk to my friends and say, "can't believe I did that! Cant believe that happened!" And then it's over with and we start talking about something else" (p. 109) |
|  | 31. Use of relaxation techniques | "Well, most times, I just walk away for a little bit, and go walk, calm myself down. And then if, like, if it's really bad, then I won't go back, but… if it's not that bad then I might go back (to the scene of where he ticced)" (p. 109) |
|  | 32. Talk about your feelings with others | "I talk to my mom because my mom had some experience with Tourette's because she almost thought she had it because when she was younger, she remembers her mother-she remembers blinking her eyes and her mother saying, "Why are you blinking your eyes?" and stuff like that... She's my go-to-guy. I talk to my mom because I feel like my dad doesn't understand me as well" (p. 110) |
|  | 33. Adopt normalizing behaviors | "Just live your life normal, I mean don't go around thinking that you've got this problem and it's not going to stop so your life's going to be bad…" (p. 113) |
|  | 34. Accept yourself | "I don't feel like I have to be in my turtle shell anymore-so, I kind of feel like I'm a part of everything and everybody else and stuff, so… And, not to let yourself feel crippled by Tourette's. Just to embrace it and be proud you have it, and to stand up and say, "Yes I have Tourette's and not to be ashamed or feel like a mutant because of it... and not to feel like you're less of a person or you're less of you. You just be yourself, and who cares if anybody sees you tic! That's how you, that's how you do it. It's who you are" (p. 113) |
| Ludlow et al. (26) | 35. Coping with children's challenging behaviour | "He had a particularly disgusting spitting tic when he was 10 or 11, and we had just no help in how to cope with that. You see him doing it out on the street and people judge really harshly." (Ellie, p.1794)  "Everyday things might get broken, like beds or tables and chairs, windows, that kind of thing due to tic related behaviours." (Vicky, p. 1794) |
|  | 36. Misconceptions and lack of understanding of professionals and the lay public | "I know it’s probably funny to watch on the TV when they’re shouting out, you know, swear words … I think some people, you know, take the mick but … it’s so tiring and when you see your son in that much pain because of it, erm, and he’s just worn out … it’s making people understand that it’s not that funny." (Rose, p. 1795) |
|  | 37. Negative experiences of children's education | "We wanted extra time for her when she come to do some exams and studying, she found it difficult to read … off a board because she were doing her eye tics and it were asked if they could make notes for Emma, you know just minor adjustments like that, which they didn’t really carry through." (Sarah, p. 1795) |
|  | 38. Support and services for families with TS | "It was very disappointing when they seemed to just offer medication or nothing. You know, their sessions were very much just listening sessions where the counsellor would just listen and repeat and make notes and that was it." (Steve, p. 1796)  "Actually it’d be nice if there was a children’s group, but because it was a mixed group with adults and children present, that didn’t really meet our needs. Erm I don’t think she would have been able to deal with some of the more extreme adults that were there." (Cathy, p. 1796) |
| Edwards et al. (27) | 39. Tic Conceptualization | "We used to call it habits. But one of our people in our school said that it might be Tourette's." (p. 41)  "something that is not your fault and… it's really hard to get to go away" (p. 41)  "I actually really don't know how to explain it [tics]" (p. 41) |
|  | 40. Awareness of Urges and Tics | Most participants reported feeling some sensation (i.e., premonitory urge) prior to their tics. Terms such as “tingle” (P4, P13), “itch” (P5), “pressure” (P4, P7), “squirm” (P12), and most commonly “a weird feeling” (P10, P14, P16) emerged during interviews. (p. 42)  "Something stuck in your throat. If you don't get it out, it bugs you a lot." (p. 42) |
|  | 41. Causes of Tics | "It just happens" (p. 42) |
|  | 42. Emotional impact | “I’m just worried that people will think I’m really weird” (p. 42)  “I feel nervous because I think it’s just gonna come over and over again" (p. 42) |
|  | 43. Social impact | “They [classmates] don’t want to hang around me because I’m weird. Like I know that there are inside jokes about my Tourette's at school and ADHD" (p. 42) |
|  | 44. Occupational (Daily Living) Impact | "It [tics] makes it hard for me to play sports because it distracts me from what I’m trying to focus on” (p. 42) |
|  | 45. Physical impact | “The snorting and sniffing sometimes makes me have nose bleeds […] and […] if I do it [clearing my throat] too much, then the throat aches after” (p. 42) |
|  | 46. Coping with TS | In terms of suppression, a 15-yearold male described how he suppresses his tics at school until the classroom is loud enough that he can “Just do it [tics] because no one will notice” (P15, p. 42)  One youth shared that when in public, he tries to hold in his tics “Because I don’t want people around to think I’m weird” (P9, male, age 13, p. 42)  With respect to disguising tics, one participant explained that she pretends to sniff while completing her lip movement tic in order to divert people’s attention away from her tic (P16, female, age ten, p. 42) |
| Cutler et al. (28) | 47. Tics get in the way and are hard to manage | "My dad didn’t just tell me to stop, he used to shout at me and make me stop it, and that’s what made me worse." (G1, P4, p. 500) |
|  | 48. TS is more than just tics | "I hate getting angry, because it’s just like, I don’t want to get angry, it’s really annoying when I get angry cause it makes me feel really sick and tired and it gives me a really bad headache and I go all red." (G2, P10, p. 500) |
|  | 49. Others not understanding involuntary behaviours | "He [Dad] used to like sort of if I ticked when I was umm. . . If I sort of ticked when he was watching TV or something he was like ‘shhh be quiet’, and I’m like ‘I can’t help it’. He never really understood that much, so . . . But he does quite a lot more now . . ." (G1, P3, p. 500) |
|  | 50. Bullying and teasing | "They like call me Tourettehead and stuff, I just end up kicking them or something, as I just get really angry and start kicking them and they start punching me, doing that back. And I end up falling out with them." (G2, P7, p. 501) |
|  | 51. Worrying about what others think | "If I do the noises they’re like ‘oh that’s Tourettes isn’t it’ and I think [few words inaudible] and I’m scared they’ll start thinking I’m weird and my relationships will go. I’m scared of that." (Pilot, P2, p. 501) |
|  | 52. Distracting and attention-consuming | "I think the reason that I can’t remember is like sometimes I don’t know what I’m doing because I’m thinking ‘oh no the Tourettes is bad ohh got to control it control it control it’ and I might ask just to go to the toilet so I can do it and then I’ve missed like 20 or 25 min of the lesson." (Pilot, P2, p. 501) |
|  | 53. Tourette syndrome is one part of who I am | "Well, I suppose if I didn’t have it I wouldn’t be myself. So that’s why, it’s part of you and part of your personality, so if I didn’t have it I wouldn’t be me. So that’s why I’ve just learnt to accept it." (G1, P4, p. 501) |
| Lee et al. (29) | 54. Uncontrollable body | "It really sucks to have TS. If I can't even sit quietly, can I still go to school? To my classmates, I'm just a freak." (Participant I, p. 283) |
|  | 55. The loneliness of not being understood | "My father often asked me to suppress tics in front of my relatives, to avoid other people asking for my situation, it did not look good, and might lose my parents’ face. I feel that I am useless; as if I have TS will have my father embarrassed." (Participant D, p. 283) |
|  | 56. Interference with academic performance | "When I am in class, my body moves around constantly, which annoys me to the point where I can't focus on reading and writing. Another reason I can't pay attention is that I worry about how my classmates perceive me and whether my behavior will interfere with their learning." (Participant B, p. 284) |
|  | 57. Compromising one's self to integrate into society | "Each time I entered a new environment, I didn't know what my classmates will think of me …, so I will observe their reactions before I decide how to interact with them." (Participant A, p. 284) |
|  | 58. Conflict between autonomy and authority | "I did not really agree with the experts’ recommendations; it's not as if they have TS, which is why I argue with my parents sometimes. They do not understand how I feel. I have my own way." (Participant B, p. 284) |
|  | 59. Helping factors in developing self-identity | "A good friend gives me the strength to control my emotions. He helps me to suppress my bad temper slowly; I am starting to discover that everybody appreciates me. I am starting to like myself and feel self‐confident." (Participant G, p. 284) |
|  | 60. Two-faced | "I feel that TS is both a stumbling block and a stepping stone. It affects my life, school, and interpersonal relationships, but comparing with people who are even worse off; I am very lucky I am willing to look at its existence from a positive point of view." (Participant B, p. 285) |
|  | 61. The power of accepting that TS is a part of you | "My parents always encourage me by the positive attitude, and they respect me, it makes me feel very warm. So, I can accept the tics even if it does not disappear." (Participant H, p. 285) |
